# The Devil in the details: The factors determining the selection of intrazonal congestion management methods across Europe

**DOI:** 10.1016/j.heliyon.2024.e26610

**Published:** 2024-02-17

**Authors:** Shilpa Bindu, Luis Olmos, José Pablo Chaves Ávila

**Affiliations:** IIT, ICAI, Comillas Pontifical University, Alberto Aguilera, 23, Madrid, 28015

**Keywords:** Congestion management, Zonal markets, Intrazonal congestion, Electricity markets, Redispatch markets, Bid filtering

## Abstract

The European electricity market is becoming increasingly interconnected, raising questions about how intrazonal congestion management, typically governed by national regulations, interacts with the coupled cross-zonal markets. A major concern is the lack of information regarding intrazonal congestion management methods. This paper addresses this issue by examining different ways in which European transmission system operators (TSOs) use third-party resources to manage congestion in short-term electricity markets. We create a decision-tree-based classification to represent all identified congestion management methods and select three cases for comprehensive evaluation using predefined assessment criteria. While doing so, we identify a trade-off between efficiency and ease of implementation. The balance between these two factors is determined by the severity of the congestion. In a severely congested grid, locational signals are critical, requiring a better alignment between the network representation in the market clearing and the physical network constraints. When the congestion is less severe, TSOs can choose other congestion management methods based on factors such as the predictability of congestion and resource availability. These findings shed light on the complexities of congestion management in an integrated European market and can inform future policy decisions.

## 1 Introduction

Congestion is a state of a transmission system characterized by one or more violations of physical, operational, or policy constraints that govern its operation under normal conditions or during any predefined contingencies [1]. According to European regulations, the responsibility of managing congestion in a grid falls upon the corresponding system operator (SO) of the grid [2]. Hence, the distribution system operators (DSOs) are responsible for congestion in distribution grids, whereas the transmission system operators (TSOs) are responsible for congestion in transmission networks. However, even within the transmission level congestion, there are additional categories that can be defined based on whether the congestion is contained within a single bidding zone or spans across two or more bidding zones. In the former case, the congestion is said to be intrazonal; in the latter case, it is said to be cross-zonal.^1^

**Fig. 1:**
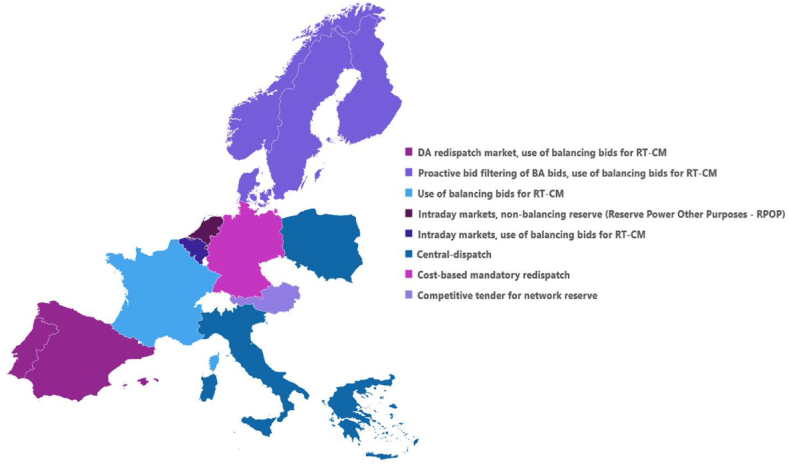
Map of intrazonal congestion management (CM) methods in selected European countries based on regulatory reviews. RT-CM stands for real-time congestion management. Note that this map may not represent all the intrazonal congestion management methods used in a country.

Congestion management involves the application of a set of procedures used by the system operators to solve or reduce the congestion in their respective grids. In Europe, the discussions on congestion management (CM) are largely limited to cross-zonal congestion management. The guidelines on Capacity Allocation and Congestion Management (CACM) set up clear requirements for calculating the available interconnection capacities and managing cross-border congestion [3]. However, the regulation on intrazonal congestion management fall under the national jurisdiction. Often, the selection of the intrazonal CM method is based on the national TSO's experience, driven by factors such as the generation mix, the system size, and the severity of congestion. Consequently, various intrazonal CM techniques are applied across Europe, as shown in Fig. 1 [[4], [5], [6], [7], [8], [9], [10], [11], [12], [13], [14], [15]]. Despite the availability of factors rationalizing the choice of an intrazonal CM in a country, the significance of such dissimilarities in an integrated European electricity market cannot be overlooked. In a meshed grid, the line between intrazonal congestion and interzonal (or cross-zonal) congestion may not always be clear; the management of one may also affect the other. Additionally, the diversity of intrazonal CM methods results in varying degrees of transparency across countries, especially regarding the locational signals provided to network users. The lack of transparency in intrazonal CM affects potential investors and limits the utilization of small, distributed resources already connected to the grid for CM services. Therefore, intrazonal CM cannot be isolated from the development of wholesale markets just because of the local nature of the congestion managed through it.

As seen in Fig. 1, many TSOs currently use balancing bids, mainly manual frequency restoration reserves (mFRR), for real-time CM. Due to this link between balancing and intrazonal CM, the ongoing integration of European balancing markets provides an opportunity to re-evaluate the CM methods used in Europe and make necessary adjustments. Identifying the types of CM methods available to the TSO is an essential first step in this process. The academic literature contains several classifications for CM methods; for relevant examples, see Refs. [1,[16], [17], [18], [19], [20]]. The classifications of European CM methods are greatly influenced by the outcomes of the ETSO Florence Regulators Meeting in 1999. There, the five main options considered for managing cross-zonal congestion were curtailments based on net transfer capacity (NTC), transmission capacity auctioning, market-splitting, redispatching, and cross-border coordinated redispatching [21]. De Vries and Hakvoort [17] structured this list of widely varying CM methods by classifying them into market-based and non-market-based mechanisms and updated the list with new CM methods. Kunz [19] proposed a similar classification based on the level of integration of transmission capacity allocation with the dispatch of the main product concerned (explicit and implicit auctions) and the criteria set for congestion alleviation (cost-based or rule-based mechanisms). Although these classifications provide a useful framework for analyzing and comparing different CM methods, we observe a greater emphasis on cross-zonal congestion. Hirth and Glismann [22] make a stride toward equal emphasis by providing an extensive overview of CM methods, classified as regulatory and physical measures. However, due to the diversity of tools in this classification, ranging from grid usage charges to nodal pricing, it is challenging to conduct a uniform assessment of options. Hence, we attempt to fill this research gap by systematically classifying intrazonal CM methods in Europe, which allows us to conduct a meaningful assessment of intrazonal CM options for future European electricity markets.

Recognizing the heterogeneity of congestion management options, we narrow down the scope of our research to short-term^2^ intrazonal CM, dealing with congestion arising in markets characterized by the following conditions: 1) TSO-owned resources (like phase-shift transformers or FACTS devices) cannot sufficiently solve all forecasted constraint violations in the system, and 2) Explicit allocation of transmission capacity is not used within a bidding zone, since this method is generally not appropriate for meshed grids. Accordingly, the main research questions that we address in this paper are the following.

1) In markets where an implicit allocation of transmission capacity is in place, how can TSOs solve dispatch infeasibilities using third-party resources?
2) What are the conditions that justify the implementation of each of the identified CM methods?

To address these questions, we start by identifying the desired qualities of a CM method. In doing so, we observe that the identified criteria can be grouped under two umbrella terms: efficiency and ease of implementation. As a next step, we conduct a thorough regulatory review to examine various intrazonal CM methods used in European countries and their coordination with the existing markets. We use a decision tree approach (for examples of similar methodology, see Refs. [23,24]) to illustrate and categorize the wide array of CM choices in the European electricity markets. This branching method results in groups, or categories, of options with homogeneous features, enabling meaningful comparisons among these groups. Further, we evaluate the identified intrazonal CM methods using the assessment framework that we define within our work. A special emphasis is given to the CM in the balancing timeframe due to the relevance of this discussion in the context of the balancing market integration. The discussion and policy recommendations from this study will be relevant for the development of the European internal electricity market, especially when focused on CM applied in zones with a high share of intermittent resources.

To the best of our knowledge, this is the first time a comprehensive classification for intrazonal CM of the type targeted (within the scope of our analysis) has been presented in the literature. The applicability of this classification method extends beyond the analysis of intrazonal CM markets. The classification tree defined can be modified for other market design analyses, such as the analysis of the coordination between local and wholesale markets. Additionally, certain forms of intrazonal CM, like mandatory and rule-based redispatch, have been extensively studied, while many others are yet to be explored in academia as possible alternatives. The lack of transparency regarding the features of intrazonal CM is a central factor conditioning previous works, leading their authors to focus on the CM methods within their respective countries that are more readily accessible. Thus, our work, presenting some of Europe's most relevant CM methods, can be deemed a starting point for identifying and evaluating different intrazonal CM methods. However, the list of options presented here is not exhaustive. By incorporating a broader range of CM practices, future studies can provide a more comprehensive evaluation of the available options, facilitating more informed decision-making in managing intrazonal congestion.

The remainder of this paper is structured as follows. Section 2 describes the assessment criteria, CM classification method, and selected case studies. Section 2.4 provides a detailed assessment of selected CM methods. Section 4 presents the discussion based on the assessment results. Section 5 shows the conclusions and main policy recommendations from this study.

## 2 Methodology

The generally accepted sequence of introducing a classification of CM methods followed by defining the assessment criteria is challenging here, as we aim to create a homogenous classification of methods with respect to the assessment criteria. We start by defining the assessment criteria and then, considering the defined criteria, create a comprehensive classification method for intrazonal CM. In the next step, we select and evaluate three different CM methods along the defined criteria. Detailed descriptions of the assessment criteria, the classification method, and the selected case studies are given in the following subsections.

### 2.1 Assessment criteria

In electricity markets where transmission capacities are not explicitly allocated, a feasible and efficient dispatch can be achieved when the effect of network constraints and losses is considered within the market dispatch process, and this effect is internalized in the resulting market prices: the ideal implementation of it being nodal pricing [25]. A simplified version of nodal pricing, called market splitting, is applied in European zonal markets to manage cross-zonal congestion [26]. However, a shift from zonal markets to a fully-fledged nodal market model at the European level would pose significant implementation challenges, not only from a technical perspective but also from political and social perspectives. Thus, when choosing a CM method, a trade-off exists between efficiency and ease of implementation.

An in-depth assessment of CM methods requires us to look more closely into the constituent qualities of an ideal CM method. Through literature and regulatory reviews, we identify certain qualities closely related to efficiency and ease of implementation^3^ [17,21,27,28]. The identified qualities form our assessment criteria as given in Table 1.

**Table 1:** The identified assessment criteria and their description.

Dimension	Assessment Criteria	Description of the criteria
Efficiency	Effectiveness	The ability of a CM method to manage and mitigate the congestion in a system without requiring additional CM methods. It can be further divided into short-term effectiveness and long-term effectiveness.
	Transparency of operation	The openness of a CM process that enables the participants to understand the outcome of the process.
	Transparency of locational signals	The clarity of the locational signals emerging from a CM process.
	Cost-efficiency	The ability of a CM method to manage congestion while minimizing the cost incurred in it. It can be further divided into short-term and long-term cost-efficiency.
	Robustness to manipulation	The ability of a CM method to minimize the possibilities for market agents to game the CM process for their own benefit, which typically leads to price distortions and inefficiencies in existing markets.
	Ease of access	A measure of the ability of a CM method not to create additional barriers of entry of the eligible participants in existing markets, nor to increase the existing barriers.
Ease of implementation	Simplicity	The measure of scalability of a system. It also concerns the easiness of understanding the rules applied within the method and the outcomes of its application in a market.
	Distributional effects	The measure of how the impact created by the implementation of a CM method is distributed among different groups of users
	Compliance with the institutional frameworks	The measure of the extent to which the adoption of a CM method can be accommodated within the existing regulatory frameworks
	Compatibility with cross-border integration	The measure of the impact of a CM method on the progress that can be made with the cross-border integration processes

### 2.2 Classification of congestion management methods

The features of a CM method and its performance depend on a series of market design choices, which are hard to represent using a simple classification model. Furthermore, for a structured assessment, the homogeneity of CM options within each group is a necessary precondition. Therefore, we use a question-based, non-binary decision tree classification, similar to the examples demonstrated in Refs. [23,24]. The tree comprises decision points, represented by nodes, and possible choices, represented by branches. From the root node, decisions are made at each branch until a terminal branch is reached, determining the CM option. It is to be noted that the developed classification is adapted to the research question that we consider here. The set of options considered in the classification proposed here is only a small part of the whole set of options available for the TSO to manage grid congestion.

**Root node selection**: Maximizing the market efficiency, which is a priority for market designers, advises managing the scarce capacity of the congested lines through capacity allocation at a global level [29]. In other words, intrazonal congestion can be decreased by better aligning the physical network layer and the network representation in the cross-zonal (first stage or primary) market clearing. However, this may make the European-wide market clearing process more complex and require sharing substantial, potentially sensitive, information. Then, two market design options are possible: 1) the overall network model is adapted to represent a larger fraction of the network congestion making use of the cross-zonal network model, thereby reducing the amount of intrazonal grid congestion, and 2) the existing cross-zonal network model is maintained while intrazonal congestion is managed through an additional CM stage.

**Fig. 2:**
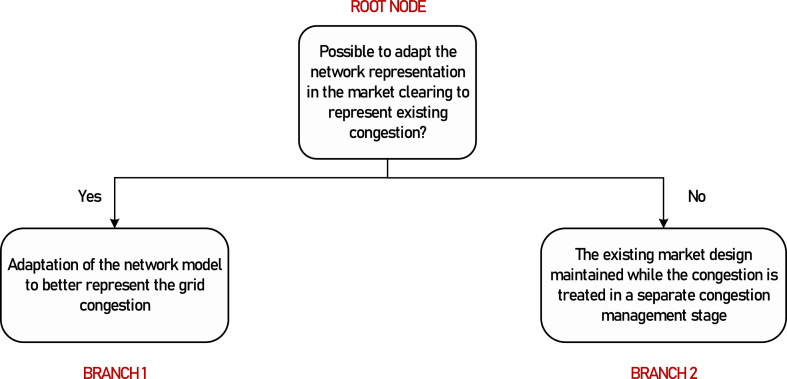
The root node and the first two branches.

The subsequent branching criteria for each branch are discussed separately, with examples of their implementation. Unless a submarket is explicitly specified, the CM method considered applies to all short-term electricity markets in the country taken as an example. The classification tree is given in Fig. 2.

**Branch 1**: Adaptation of the network model to better represent the prevailing grid congestion.

In European electricity markets, the primary method through which the network is adapted for managing severe intrazonal congestion is bidding zone reconfiguration (BZR). The BZR process redefines the existing zones through splitting, merging, or a combination of both to represent the congestion better. This process is conducted by ACER and ENTSO-e every three years [3,30]. A shift to a locational marginal pricing (LMP) model can be considered an extreme form of BZR.

**Fig. 3:**
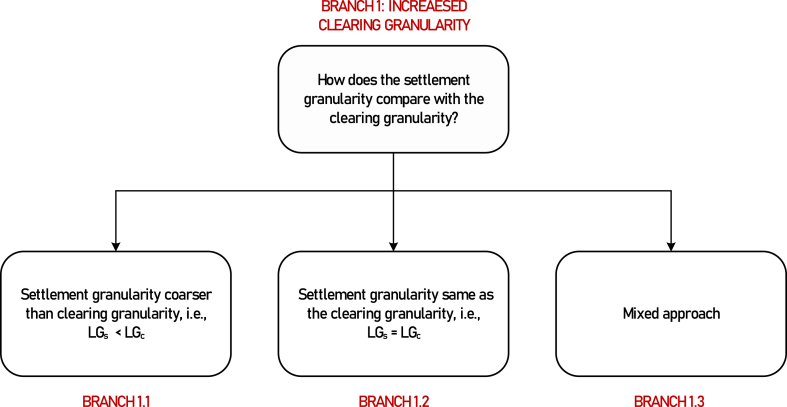
Classifications within Branch 1 (increased clearing granularity).

**Motivation for further branching:** When a large zone, like a single national zone, has to be divided into smaller zones to manage intrazonal congestion, consumers from one zone situated in one part of the country may be subject to higher prices than consumers located in another zone, situated elsewhere in the country, creating distributional effects within a country and reducing the public acceptance. A case in point is Germany, where, despite evidence indicating the pertinence of splitting the single existing bidding zone into multiple smaller zones, political concerns have emerged as the key barrier to the new zones adoption [[31], [32], [33]]. To overcome resistance, it is possible to modify one or more market design elements to address the socio-political concerns without altering the newly defined bidding zones.^4^ Thus, in Italy, despite the existence of several bidding zones, a single national price called PUN (*Prezzo Unico Nazionale*) is applied to consumers, while the generators, pumped storage, and cross-border consumers pay or receive their corresponding zonal price [34,35]. Another lesser-known example is that of the European central-dispatch markets, where the network is represented with a finer granularity in the balancing market clearing to obtain a feasible dispatch, but the settlement is done at a zonal or national level [36,37]. Considering these previous options, we apply the following branching criteria to further classify those CM methods, as shown in Fig. 3.

**Further branching criterion**: Relationship between locational network granularity considered for clearing (*LG*_*c*_) and that considered for settlement (*LG*_*s*_)

**Branch 1.1 (Terminal)**: Clearing granularity same as settlement granularity (*LG*_*c*_*= LG*_*s*_).

Example: Zonal markets of Sweden and Norway, nodal markets (not currently present in Europe) [33]

**Branch 1.2 (Terminal):** Clearing granularity finer than settlement granularity (*LG*_*c*_ *≥ LG*_*s*_).

Example: Central-dispatching balancing markets of Greece [36]

**Branch 1.3 (Terminal):** Mixed approach whereby different groups of market players are settled at different granularities.

Example: Italian electricity markets^5^ [34]

The summary of options discussed within Branch 1 is given in Table 2.

**Table 2:** Summary of options available for increasing the granularity, along with the examples for their implementation in the European markets.

Branch	Description	Example
1.1	Clearing granularity same as settlement granularity	Zonal markets of Sweden and Norway, Nodal markets of New Zealand
1.2	Clearing granularity finer than settlement granularity	Central-dispatching balancing markets in Italy, Greece and Poland
1.3	Mixed settlement	Italian markets (generator pays the zonal price and demand pays a national price)

**Branch 2:** Maintaining the existing markets while addressing congestion separately (in an additional stage)

**Fig. 4:**
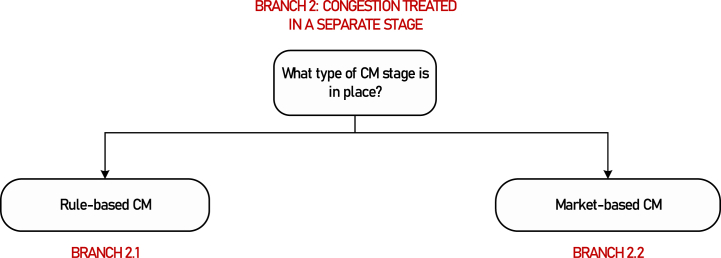
Classification of second-stage congestion management with respect to the type of CM stage.

Even though the detailed grid representation in market clearing is required for delivering efficient dispatch and accurate locational signals, the ongoing market integration processes at the European level can create additional barriers to considering a granular European network. Detailed grid data is sensitive information, preventing some countries from sharing it within a European platform. In such cases, a two-stage market clearing approach can be adopted. First, at the European level, the market clearing is performed using a simplified network model, calculating cross-border exchanges and flows, as well as the congestion rents resulting from valuing imports and exports at the marginal price computed for each zone as a byproduct of the European market clearing process. Then, within each zone, in a second stage, the market is cleared using a more detailed grid representation and fixed cross-border flows as obtained from the previous stage. These two options are shown in Fig. 4.

**Motivation for further branching:** CM, as a separate process, can be arranged as a rule-based or a market-based mechanism.^6^ As these two types of schemes are largely different, they should be considered separately.

**Further branching criterion**: Type of the CM stage.

**Branch 2.1**: Rule-based CM.

The TSO can procure CM resources through a predefined procedure outside the existing market. These rules can be simple to expedite the process of managing congestion and producing a feasible dispatch. Whenever the level of competition is not high enough, market-based approaches should not be applied. It could be argued that market-based approaches can be combined with market power mitigation mechanisms. However, proving the exercise of market power is challenging, especially in markets with high concentration like congestion management and balancing markets.

**Fig. 5:**
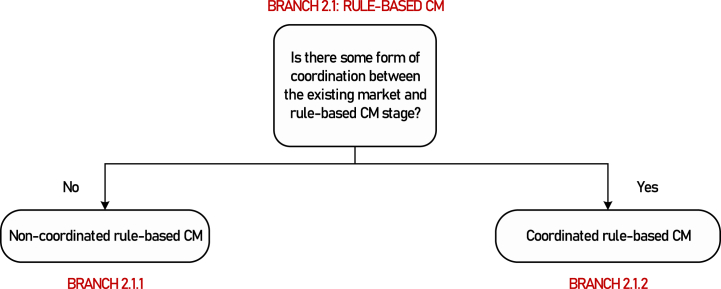
Classification of rule-based CM based on coordination with the existing markets.

**Motivation for further branching**: Within a rule-based process for managing the congestion arising from a market, the TSO can still use the bids from this market. We refer to this model as coordinated with the existing market. On the contrary, when the CM stage does not consider bids from the existing market and, instead, selects the CM resources to use based on information sourced from elsewhere (for example, from a separate bidding stage, if market-based, or from registration data, if non-market-based), we refer to this as a non-coordinated process. Fig. 5 depicts these two options.

**Further branching criterion**: Existence (or not) of some coordination of the rule-based CM mechanism with the existing market.

**Branch 2.1.1(Terminal)**: Non-coordinated, rule-based CM.

Example: load curtailment.

**Branch 2.1.2**: Coordinated, rule-based CM^7^

**Fig. 6:**
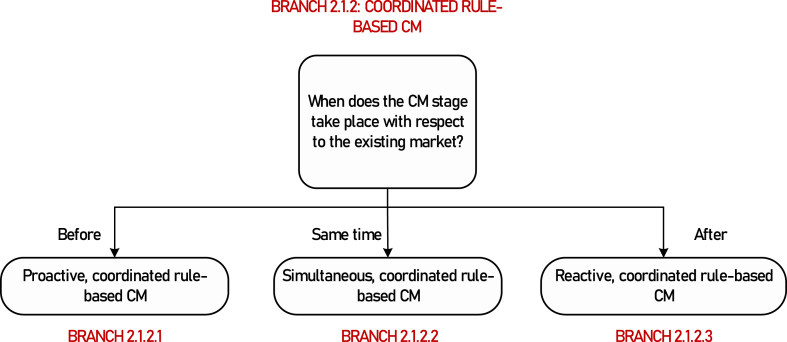
Classification of CM with respect to the timing of the existing market.

**Motivation for further branching**: The level of performance of a market-based CM mechanism depends on the chronological sequence considered for the CM mechanism and the existing market. If the CM process is proactive, i.e., it takes place before the clearing of the existing market, its effectiveness and cost-efficiency will depend on the ability of the TSO to accurately forecast the power flows that will result from the market. However, if the CM process is reactive, i.e., if it takes place after the existing market, the TSO knows the power flows resulting from the market giving rise to congestion, which is likely to increase the effectiveness and efficiency of the CM method. Hence, the timing of CM relative to that of the market timing^8^ creating the congestion to manage is a relevant criterion for assessing the performance of a CM method. Fig. 6 presents the three options within coordinated rule-based CM.

**Further branching criterion:** Timing of CM relative to that of the existing market.

**Branch 2.1.2.1(Terminal)**: Proactive, coordinated, rule-based CM.

Example: Proactive bid filtering in Nordic balancing markets [38]

**Branch 2.1.2.2(Terminal)**: Simultaneous, coordinated, rule-based CM.

Example: Coordinated CM and balancing clearing in France [39]

**Branch 2.1.2.3(Terminal)**: Reactive,^9^ coordinated, rule-based CM.

Example: Use of balancing bids for redispatch in Spanish markets [8]

**Branch 2.2**: Market-based CM.

**Fig. 7:**
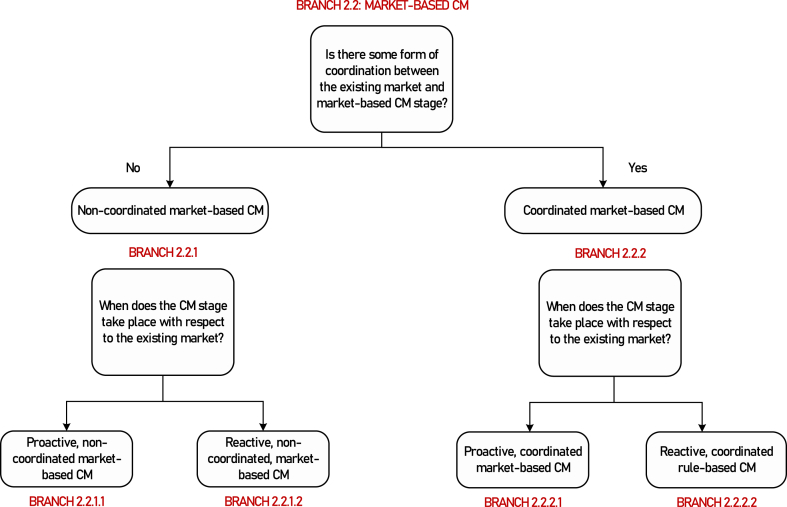
Classification of market-based CM methods.

**Motivation for further branching**: We repeat the same branching criteria used in Branch 2.1.1 to obtain the following terminal branches. The full classification of market-based CM is given in Fig. 7. Additionally, the overall classification is tabulated in Table 3.

**Table 3:** Summary of options available within separate management of congestion outside the market, along with examples of their implementations in the European market.

Branch	Type of CM	Coordination with existing market	Timing of the CM wrt existing market	Example
2.1.1	Rule-based	Non-coordinated	–	Load curtailment
2.1.2.1	Rule-based	Coordinated	Proactive	Proactive bid filtering in Nordic balancing markets
2.1.2.2	Rule-based	Coordinated	Simultaneous	Coordinated CM and balancing clearing in France (to be replaced by bid filtering)
2.1.2.3	Rule-based	Coordinated	Reactive	Use of balancing bids for redispatch in the Spanish markets
2.2.1.1	Market-based	Non-coordinated	Proactive	Preventive local CM markets (OneNet project)
2.2.1.2	Market-based	Non-coordinated	Reactive	Spanish DA redispatch markets
2.2.2.1	Market-based	Coordinated	Proactive	Theoretical
2.2.2.2	Market-based	Coordinated	Reactive	Curative local CM markets (OneNet project)

**Branch 2.2.1.1(Terminal)**: Proactive, non-coordinated, market-based CM.

Example: OneNet preventive CM markets [40]

**Branch 2.2.1.2(Terminal)**: Reactive, non-coordinated, market-based CM.

Example: Spanish day-ahead redispatch markets [8]

**Branch 2.2.2.1(Terminal)**: Proactive, coordinated, market-based CM.

Example: Proactive bid filtering markets based on voluntary participation and transparent rules (theoretical)

**Branch 2.2.2.2(Terminal)**: Reactive, coordinated, market-based CM.

Example: OneNet curative CM market [40]

**Fig. 8:**
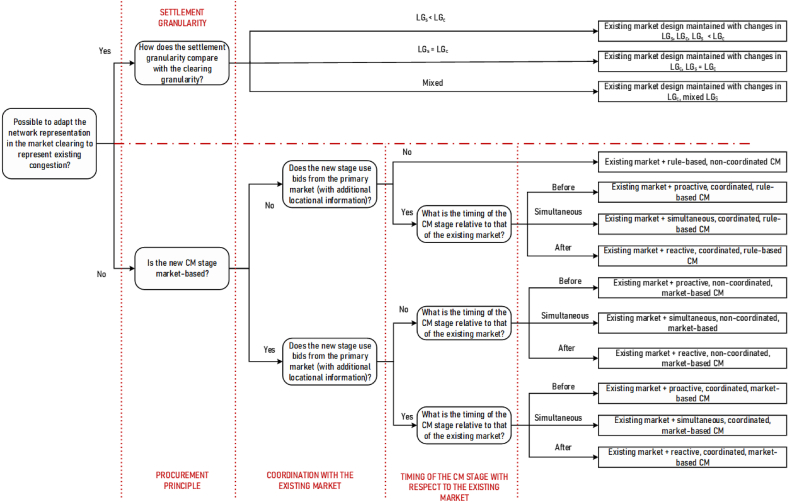
Classification of options available for the TSOs to manage intrazonal congestion in short-term electricity markets in Europe.

The overall classification tree is presented in Fig. 8.

### 2.3 Relationship between branch 1 and branch 2

Although CM options within Branch 1 and 2 represent two different families of approaches, both use the some variants of the optimal power flow (OPF) to manage congestion (in the first stage in Branch 1 and the second stage in Branch 2). Hence, theoretically, the dispatch results produced by both families of approaches could be the same provided some conditions are met. Next, three instances of specific congestion management arrangements leading to the same dispatch as the fully integrated CM scheme are discussed.

1) If the market operator considers all the resources that have participated in the first-stage market when applying rule-based reactive CM (Branch 2.1.2.3) right after this first-stage market (so that there is enough ramping time for all resources to be mobilized), and the TSO considers a cost-minimization optimization function in the CM stage (instead of, for example, minimizing the deviations from the previous market positions), the final dispatch resulting from this two stage process should be the same as that of an integrated market.
2) If all the participants in the existing, first-stage, market participate in a second stage reactive, coordinated, CM market (Branch 2.2.2.2) that takes place right after the existing market, and the TSO considers a cost-minimization optimization function in this second stage, the dispatch results from this should be the same as those of a fully integrated system.
3) If all the participants in the existing, first stage, market participate in a second stage, reactive, non-coordinated CM market (Branch 2.2.1.2) that takes place right after the existing market, using the same bids as in the existing market, the conditions applying here would be the same as those in 2). In this case, the resulting dispatch could be the same as that resulting from a fully integrated CM market.

The probability that any of these three sets of conditions are met in a wholesale market like the European DA market is very low. Hence, the dispatch results from Branch 2 are almost always less efficient than those from Branch 1, i.e. they would be suboptimal.

### 2.4 Evolution of the intrazonal congestion management methods in Europe

**Fig. 9:**
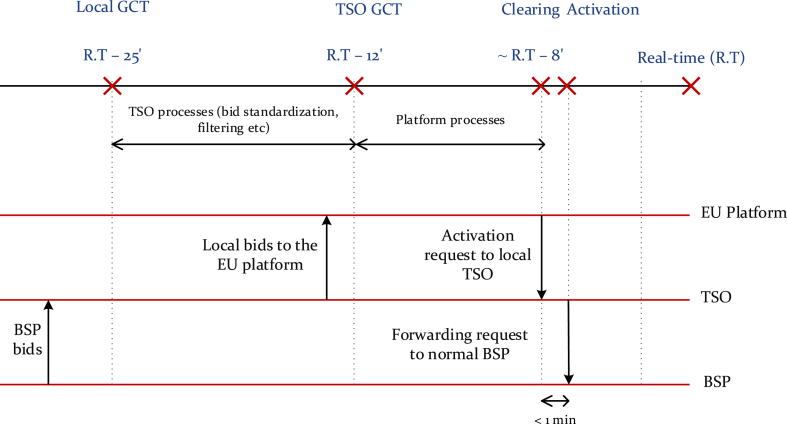
Time schedule for the mFRR market.

Even though the treatment of intrazonal CM is under the national jurisdiction, the regulatory changes taking place at the European level have significant effects on the intrazonal CM methods used in European countries. A good example of this is related to the practice of using balancing bids for managing intrazonal congestion. Many TSOs use balancing energy bids to manage real-time congestion, as shown in Fig. 1. After the gate closure time (GCT) of the balancing energy markets (⁓1 h before delivery), TSOs clear the market and check the effect of the cleared balancing bids on their network. If deemed necessary to address congestion, they conduct a redispatch process considering the available balancing bids before sending activation requests to the corresponding balancing service providers (BSPs) [13]. However, once the European balancing energy exchange platforms are fully operational, these platforms will select the mFRR bids to be activated just before real-time, leaving the TSO less than 1 min to forward the activation requests to the BSP, as shown in Fig. 9 [41,42]. Within that short period of time, the TSO will need to analyze the effect of the mobilization of the resources corresponding to the selected balancing bids on the system constraints and redispatch these and other resources, if needed. Due to the highly time-sensitive nature of this process, the Electricity Balancing Guidelines (EBGL) recommend that the TSOs filter the balancing bids received from their control areas before forwarding them to the European platforms [43]. This approach allows TSOs to proactively identify any potential congestion due to the activation of certain bids and to mark these bids as unavailable for cross-border exchange of balancing energy. Hence, a shift from reactive CM to proactive CM can be seen in the balancing markets as a result of the regulation put in place.

Another piece of legislation affecting the evolution of CM methods is the EU regulation 2019/943, recommending market-based methods for redispatching [44]. This article of legislation states that redispatching (or reactive CM methods) shall be based on market-based mechanisms. Exceptions are allowed only if market-based mechanisms are unavailable, there is a lack of competition, or the congestion occurring is highly predictable and leads to gaming opportunities. As a result of this, a shift from rule-based CM to market-based CM could be expected in the European DA and ID markets.

In the medium term (or even long term, as some reconfiguration processes take years to get the approval), the preferred method for intrazonal CM in Europe is bidding zone reconfiguration to avoid, or decrease, intrazonal congestion [3]. Another market model that also uses highly granular network data for market clearing, called the central-dispatching model, is only allowed in certain countries [45]. Therefore, in principle, any change in clearing granularity would result from the bidding zone reconfiguration process.

Even though co-optimizing the procurement of energy and system services is an efficient design choice, while assessing each method individually, it is incompatible with cross-border integration due to computational complexity and data protection rules [41]. Hence, simultaneous, coordinated procurement of products is not expected in the near future.

### 2.5 Selected case studies

A thorough analysis of all the CM methods identified above would exceed the article length limitations; hence, we select for their assessment examples of three distinct CM methods (terminal branches) that, a priory, make promising options for future implementation in European electricity markets. Their selection is motivated by the discussions given in Section 2.4.

• **Branch 1.1** Adaptation of the network model to better represent grid congestion and considering the same clearing granularity as the settlement granularity – Splitting of bidding zones
• **Branch 2.1.2.1** Proactive, coordinated, rule-based CM – Proactive bid filtering
• **Branch 2.2.1.2** Reactive, non-coordinated, market-based, CM – Redispatch markets.

The following section briefly discusses selected CM mechanisms and their relevance in future electricity markets.

#### 2.5.1 Adaptation of the network model to better represent grid congestion and considering the same clearing granularity as the settlement granularity – splitting of bidding zones

Internally well-meshed areas can be considered super-nodes for CM purposes if no considerable congestion is observed within these areas and the dispatch within each does not result in loop flows outside the zone [25]. When these conditions are not met, the existing bidding zones should be redefined through splitting or merging of bidding zones or a combination of both. The regulatory foundation of the bidding zone delimitation was established in 2015, by the Capacity Allocation and Congestion Management Guidelines (CACM). These guidelines set the requirements to be met when revising the existing configuration of bidding zones in Europe every three years [3,30]. Currently, most European countries feature a single national bidding zone whose borders are the national boundaries. Exceptions are Norway, Sweden, Denmark, and Italy, which have multiple bidding zones within the country. Within the reconfiguration process, only splitting bidding zones within a country, or merging bidding zones within a country, is permitted [46]. The full or partial alignment of zones with the political borders avoids the formation of price regions spanning several countries, which can create operational challenges.

A possible direction for future market developments involves considering different levels of granularity in different short-term markets. At the moment, any increase or decrease in the granularity of the network model considered in markets, through bidding zone reconfiguration, applies equally to all markets. Poland is currently considering the implementation of a nodal balancing market that could co-exist with other currently existing short-term electricity markets (like the DA one), leading to speculation about the potential hybridization of market models [41,47].

#### 2.5.2 Proactive, rule-based, coordinated congestion management: bid filtering

Proactively managing congestion is useful when the TSO does not have enough time available to follow a reactive approach. This is the case of the European balancing market, where the TSOs receive the activation requests issued by the European balancing platform very close before the delivery period (as illustrated in Fig. 9). Although the regulation permitting the marking of unavailability is associated with balancing markets, this concept can easily be applied to any market close to real-time [43]. The mechanism used by the TSOs to mark the unavailability of bids is within the national scope and is not always transparent to market players [48]. Certain computational approaches for bid filtering processes can be found in Refs. [49,50]. According to these works, the TSO simulates different exchange scenarios and iterates the power flow in its grid with different combinations of bids. The bids that do not cause congestion in any scenario can be forwarded to the platforms, but the availability of the bids that may cause congestion in some of the simulated scenarios will be determined by the filtering criteria defined by the TSO.

#### 2.5.3 Market-based, reactive, non-coordinated congestion management: redispatch markets

Reactive congestion management markets, or redispatch markets, are marketplaces for procuring resources to address the network infeasibilities resulting from a previous market while maintaining the balance between generation and demand. Those market units that have flexibility available to increase or decrease their output with respect to their scheduled market position submit the corresponding bids to the redispatch market. Taking these as an input, the TSO, then, selects the most cost-efficient combination of bids that solves the congestion in the grid.

The dedicated day-ahead CM market in Spain is a perfect example of a reactive, non-coordinated CM market. Spanish day-ahead market is followed by a CM market where market players bid separately. The TSO procures CM resources in two steps. First, the TSO addresses the constraint violations arising from the DA market by adjusting the generation output levels. In doing so, the demand-generation balance is affected. Thus, in the second step, the TSO uses the CM bids from the other end of the congested line to rebalance the system [8]. Despite the prevalence of the use of balancing bids for real-time CM through rule-based mechanisms, to the best of our knowledge, non-coordinated redispatch markets in the balancing timeframe have not been implemented in Europe.

## 3 Assessment of selected congestion management methods

Combining multiple CM methods is possible within any system. For instance, Nordic markets utilize proactive bid filtering and the rule-based use of mFRR bids alongside multiple bidding zones (Nordic Operations Development Group 2021). Assessing the individual effects of each method can be challenging due to their overlapping and interactive nature. Although we qualitatively evaluate each method in isolation here, in practice, it is highly unusual for a single intrazonal CM method to be implemented alone.

### 3.1 Adaptation of the network model to better represent grid congestion and considering the same clearing granularity as the settlement granularity – splitting of bidding zones

**Effectiveness:** If bidding zones are reconfigured to represent intrazonal congestion accurately, they can effectively mitigate congestion in the short term without requiring additional resources for managing congestion. In the long term, the investments will respond to the price differentials between the zones and move to the higher-priced zone. However, BZR is not a dynamic process in Europe, as the zones are only reviewed every three years [3]. This can result in suboptimal zones if there are changes in the volume and location of intrazonal congestion between the review periods. Additionally, the constraint to define bidding zones that are a subdivision of political ones, instead of considering uniform price contours, has been criticized in the literature, as the resulting bidding zones may diverge from the most optimal configuration for managing the intrazonal congestion or an excessive number of zones may need to be defined [[51], [52], [53]].

**Transparency of operation:** The BZR process and the cross-zonal market operations follow the transparency requirements set at the European and national levels.

**Transparency of locational signals**: BZR can provide transparent locational signals corresponding to the granularity of the new zones. If a transmission line is frequently congested, leading to a zonal split along that line, the potential investors receive locational signals corresponding to the price differential between the two zones. Analogously, if the market provides node-level signals, investors may be motivated to locate resources in high-price nodes.

**Cost-efficiency:** BZR should reduce the amount of intra-zonal congestion, which is not addressed through a market-based, integrated CM scheme in the existing market. Since intrazonal congestion will be managed separately within each zone in a non-system-wide-coordinated manner, reducing intrazonal congestion should increase cost-efficiency in the operation time frame. Besides, when bidding zones are reconfigured to reflect congestion, there will be an associated redistribution of costs and revenues. Compared to the prices before reconfiguration, consumers in the generation-abundant zones will be applied lower prices, and those in the generation-scarce zones will be applied higher prices. A similar redistribution effect can also be seen in the generators' revenues, consumption, and storage facilities. These robust short-term price signals guide future investments in constrained regions, increasing long-term cost-efficiency. While that argument is logical [54], demonstrate that, although bidding zone-splitting reduces CM costs in the short term, social welfare may decrease in the long term, owing to the excessive accumulation of investments in high-price regions. Dynamic zone reconfiguration has the potential to address this issue, but it may lead to a reduction in the stability of the zones.

**Robustness to manipulation:** While maintaining large zones can result in higher CM costs, splitting bigger zones can result in higher levels of market concentration [52]. It is also argued that some market players may strategically bid to trigger market-splitting. However, in a strongly meshed grid, like that in most areas in Europe, the neighboring zones strongly influence the trades within a bidding zone, making strategic bidding less likely [51]. Moreover, higher market concentration does not always correlate with higher market power [[55], [56], [57]].

**Ease of access:** BZR, or in particular, the splitting of zones, does not directly create entry barriers. Still, when a large zone is divided into smaller ones, the area available for grouping the units decreases, limiting the self-balancing and aggregation possibilities within portfolios managed by market players. These limitations to portfolio bids may affect large power producers to a larger extent than small participants. Without self-balancing possibilities, large producers might be obliged to participate in the intraday markets to adjust their commercial positions, thereby increasing intraday liquidity [58]. This can be a desirable effect for the system. However, aggregation is a condition that facilitates the integration of distributed resources into the existing wholesale markets, especially when the minimum bid size requirements in the market are high. Looking at the existing nodal markets, one can see that market designers can make provisions to support aggregation. CAISO, a nodal market in the US, addresses the aggregation issue by developing custom load aggregation points (CLAP) through which the demand-side consumers can aggregate their load across multiple nodes [59].

**Simplicity:** When the number of zones inside a country increases substantially, the allocation of cross-zonal transmission capacities becomes an issue of major consideration. If the defined bidding zones are large enough, the adjustments to be made to the national and European platforms may not be relevant. However, when a shift to a nodal scheme is made, the European grid model comprises more than 25,000 transmission nodes^10^, which can create severe computational challenges [61].

**Distributional effects:** Adopting a granular market design can elicit different public reactions depending on the country of implementation. For example, Sweden could split the single national zone into smaller zones with relative ease due to the positive experiences of their Nordic neighbors with market-splitting [62]. However, as discussed in Section 2.2, in Germany, political concerns have deferred a much-needed bidding zone reconfiguration. A differential pricing scheme, similar to the Italian PUN pricing (single national price for demand), could improve the acceptance of more granular network models in the market. However, the growing significance of prosumers and storage devices requires more regulatory interventions to ensure fair and efficient pricing in such a differential (hybrid) pricing system.

**Compliance with the institutional framework:** Since liberalization, European electricity markets have always used a zonal model. Therefore, the institutional and organizational frameworks are designed specifically for zonal models. If large enough zones can be defined, they can be accommodated within the existing framework without significant changes. However, if the number of zones is largely increased, such as when a nodal model is adopted, high transition costs would be incurred due to the resulting need for considerable changes in the market design, infrastructure, and operational procedures [63,64].

**Compatibility with cross-border integration:** Zonal reconfiguration is compatible with European institutional frameworks and integrated market platforms. However, it may have implications for cross-border exchanges. In a meshed network, like the Central-Western European, zonal reconfigurations will have spill-over effects in the neighboring areas. Authors [31] study the market-splitting of Germany into northern and southern areas and observe that the consumer welfare in the countries closely connected to wind generation abundant northern Germany increases, whereas the consumer surplus in the countries directly connected to the demand-intensive south decreases. Authors [65] also came to a similar conclusion after studying market-splitting in Germany. Furthermore, sub-optimal bidding zone configurations can also create unscheduled power flows between zones, reducing the margin reserved for cross-border exchanges [64]. Therefore, any changes in the market design should be carefully coordinated with the neighboring countries to minimize the resistance of some countries to facilitate this reconfiguration process.^11^

### 3.2 Proactive rule-based coordinated congestion management: Proactive bid filtering

**Effectiveness:** Proactive bid filtering removes the bids that can cause congestion, increasing the feasibility of the results. However, its effectiveness depends on the accuracy of the power flow simulations [41,66]. Systems using proactive CM methods may still require additional CM measures to manage real-time deviations and unscheduled flows. Hence, the short-term effectiveness of a proactive method tends to be lower than the reactive CM, where there is a degree of certainty on the power flows resulting from the market dispatch. The long-term effectiveness of a proactive bid filtering method is determined by the accuracy of predictions and the transparency rules (discussed below).

**Transparency of operation:** In the bid filtering mechanism currently used by the TSOs, transparency of operation enabling market participants to understand price formations is often lacking. A potential reason for the reduced transparency is the use of complex algorithms to predict congestion resulting from real-time activations. Publication of data providing predicted congestion and motivation for bid filtering can improve the transparency of this mechanism.

**Transparency of locational signals:** If the bid filtering process used in the balancing markets is considered an example, the filtering takes place between the local gate closure time for the balancing service providers (BSPs) and the platform gate closure time for the TSOs. During this time, TSO can mark a bid as unavailable for various reasons, such as to avoid possible internal congestion or to maintain a pool of resources for redispatch or direct activation [43]. The reason for the ‘unavailable' tag is unknown to the market players, eliminating any locational signal arising from this process. Similar to the proposal for increasing transparency of operation, an ex-post publication of forecasted congestion data and motivation for bid filtering can improve the visibility of locational signals.

**Cost-efficiency:** In markets where bid filtering is currently used, the filtered bids are not remunerated even if the predicted congestion does not materialize in real time [48]. Removing bids that would have been activated from the merit-order list can potentially increase the cost of providing the corresponding service (balancing, in the example), increasing the operational costs. Besides, this would create price distortions, as the higher prices resulting from not considering the filtered bids reflect the cost of proactive CM rather than the true marginal cost of balancing that would have resulted from considering the most efficient bids that are compatible with grid constraints. Such price distortions negatively affect the overall cost-efficiency of the system.

**Robustness to manipulation:** As discussed in the previous subsection, proactive bid filtering is a non-remunerated, rule-based, CM method. Therefore, this method leaves less room for agents to manipulate the resulting dispatch and prices in their own benefit, since, under this scheme, the auctioneer does not make use of any information provided by agents to decide whether to consider them in the subsequent market.

**Ease of access:** Proactive bid filtering does not create any discriminatory conditions for participation. However, it can be argued that certain participants located along a congested line can be repeatedly filtered out, limiting their market participation.

**Simplicity:** Even in systems with stable congestion levels, the bid filtering algorithm can be complex; an example of such an algorithm can be seen in Ref. [50]. Replicating and scaling up this algorithm for a system with severe and unpredictable congestion can be computationally challenging.

**Distributional effects:** Bid filtering does not create differential prices for the consumers, but such schemes can be discriminatory towards the market players who are repeatedly filtered out even if the predicted congestion does not materialize in real-time.

**Compliance with the institutional framework:** Apart from the requirement to develop a new algorithm for the bid forwarding process, severe changes in institutional or organizational setup are not expected from its adoption.

**Compatibility with cross-border integration:** The electricity balancing guideline (EBGL) proposes bid filtering to proactively manage congestion from balancing energy activations [43]. Such a scheme would allow the TSO to have some level of control over real-time congestion while maximizing cross-border exchange opportunities instead of reducing the NTC values to minimize intrazonal congestion. Hence, a proactive bid filtering scheme can positively impact the market if able to accurately identify those bids that are contributing to the increase in congestion while being less competitive than others also creating congestion and competing with the former for the use of the scarce network capacity.

### 3.3 Market-based, reactive, non-coordinated congestion management: redispatch markets

**Effectiveness:** In the short-term, redispatch markets can be effective as the TSO has full certainty on the power flows from a congestion-causing market. However, the long-term effectiveness is determined by the transparency and the accuracy of the locational signals. When a redispatch market takes place after an existing market, say primary market, whose market dispatch creates congestion, theoretically, the most accurate results will be obtained when all the participants in the primary market are also considered in the redispatch market.^12^ In reality, only a set of participants from the primary market participate in the redispatch market, contributing to the price signals.^13^ Besides, redispatch markets can be easily manipulated by the few market players in congested regions, further decreasing the accuracy of the price signals. Hence, redispatch markets cannot effectively manage congestion in the long term.

**Transparency of operation:** Market-based procurement, in general, is more transparent than rule-based procurement. However, the national regulations and market design will determine the transparency level of individual redispatch markets.

**Transparency of locational signals:** Within a redispatch market, prices computed in the CM process are only applied to those bids that are dispatched to manage congestion, while those bids originally dispatched within the existing market are applied to the original prices computed in this market. Given that the prices applied on the majority of transactions, those arranged in the existing market, do not reflect congestion, they are not transparent in this regard. However, incorporating a market-based approach in the redispatch process and making the resulting prices visible to all market participants can increase the transparency of the resulting locational signals w.r.t. a case where non-market-based methods are applied.

**Cost-efficiency:** Redispatch markets have been criticized heavily in the academic literature due to the high costs associated with redispatch. This is indeed true in certain cases, as redispatch prices are only applied on redispatched agents and not on others sharing location with the former. Unfair discriminatory prices induce strategic behavior by many agents artificially manipulating their bids, which would no longer reflect the costs they would incur in providing the corresponding service. This is likely to affect the merit order and the resulting dispatch, thus increasing the cost of the service provision. Besides, it would create misleading long-term signals reducing the long-term cost-efficiency of the system [67].

However, at the same time, examining the resources available for redispatch partially reveals the rationale behind the elevated prices produced in these markets. Many European TSOs currently use balancing bids for real-time CM, as shown earlier in Fig. 1. Only a limited number of resources are eligible to provide balancing services complying with the corresponding strict technical requirements. Now, within this limited pool of expensive resources, the TSOs have to select bids compatible with the network constraints. For the upward direction, the resulting marginal bid will probably be more expensive than the marginal in all the previous markets, making up for the opportunity costs. Similarly, in the downward direction, the participants may try to reduce the payment they have to make to the TSO, bidding much lower than their marginal costs.

Apart from the impact on the cost of service, whenever congestion is created in a market for a certain service (e.g., day-ahead market) is addressed through a redispatch market considering the bids issued to provide another service (e.g., balancing) as it is the case in some systems, the prices resulting from the market for the second service do not reflect the marginal conditions for the provision of this service (balancing). This creates perverse incentives affecting the short- and long-term decisions by agents and the corresponding operation and investment costs in the short and long term. Therefore, evaluating the cost of congestion requires an assessment of the position of redispatch markets within the sequence of the existing markets.

**Robustness to manipulation:** Redispatch markets are largely subject to the manipulative behavior of agents, i.e., participants may exploit historical market data and intentionally trigger congestion, as a set of market participants have incentives to bid away from their marginal costs in the existing market and receive higher revenues. This form of market abuse, called inc-dec gaming, is discussed as one of the main disadvantages of redispatch markets [68,69]. On the other hand, when the alternative for a redispatch market is bidding zone reconfiguration, a concentration of units from a few owners can aggravate the possibilities of market power abuse, increasing the system costs in all the short-term markets. However, as discussed under this same criterion in zonal reconfiguration, the mere existence of market concentration does not equate to market arbitrage, especially in heavily interconnected grids.

**Ease of access:** Redispatch markets for managing congestion from a primary market do not create serious entry barriers to the existing markets. In fact, CM markets, both at transmission and distribution levels, are explored in many flexibility market pilots as a potential revenue stream for small-sized distributed resources [40,70].

**Simplicity:** The simplicity of the market rules and clearing algorithms depends on the market design features. Assuming a redispatch market similar to the Spanish DA CM market, the design is easily scalable and simpler to understand than a very complex, albeit efficient, network-constrained zonal market with a large number of zones.

**Distributional effects:** Redispatch markets are not expected to create discriminatory prices for consumers. However, redispatch markets can result in the differential treatment of units located along congested lines by providing additional revenue streams and motivating them to engage in market arbitrage attempts in the primary market.

**Compliance with the institutional framework:** EU regulations recommend using market-based redispatching except when market-based procurement of CM resources is not competitive or efficient [44]. However, some member states have previous experience operating a redispatch market, like Portugal and Spain, whereas a few others, like Germany, use a rule-based redispatch. Thus, based on the status quo, the ease at which the institutional frameworks can be set up to implement a redispatch market can differ.

**Compatibility with cross-border integration:** As redispatch happens after the main market, whether day-ahead, intraday, or balancing, it does not directly affect the participation of market participants in any European markets, nor does it create any additional implementation issues for European market platforms.

## 4 Discussion

This paper presents a systematic classification of intrazonal CM techniques and an assessment framework that can be used to evaluate them. We evaluated three different cases of intrazonal CM and provided their detailed assessment. The results are summarized in Table 4. While assessing all available options for CM, discussed and not discussed here, we observe that the choice of a CM method is subject to a trade-off between efficiency and ease of implementation. A major factor in determining the right balance between the two is the severity of congestion.^14^ When congestion in a power system is deemed severe, the most appropriate course of action (and approved by regulation) is to adapt the network model to precisely represent the underlying structural congestion in the procurement process.

**Table 4:** Summary of the evaluation of the three congestion management methods. Positively evaluated criteria are marked with a positive sign (+), and negatively evaluated ones are marked with a negative sign (−). NA – Not Applicable.

	Splitting of bidding zones	Proactive bid filtering	Redispatch markets
**Effectiveness – short-term**	**+**	**+**	**+**
**Effectiveness – long-term**	**+**	**-**	**-**
**Transparency of operation**	**+**	**-**	**+**
**Transparency of locational signals**	**+**	**-**	**-**
**Cost-efficiency**	**+**	NA	**-**
**Robustness to manipulation**	**+**	**+**	**-**
**Ease of access**	**-**	**+**	**+**
**Simplicity**	**+**	**-**	**+**
**Distributional effects**	**-**	**+**	**+**
**Compliance with the institutional framework**	**+**	**+**	Depends on the existing national regulations
**Compliance with cross-border integration**	**+**	**+**	**+**

In the European case, market coupling further complicates the possibility of adapting the network representation to provide a European-wide efficient dispatch and efficient and transparent locational signals. Factors such as the sensitivity of grid data and the computational complexity of clearing the market for a large region challenge the adoption of a locational pricing system. Under such conditions, theoretically, it is possible to clear the market in two stages. In the first stage, a simplified network is considered, complying with the regulations of the EU. In the second stage, a detailed network representation is considered together with cross-border exchange values computed in the first stage. This allows complying with the national regulation regarding the management of grid congestion and computation of a feasible dispatch. Given that those schemes providing locationally differentiated prices within zones are not yet implemented in Europe, these schemes are not considered for the study at the moment.

**Fig. 10:**
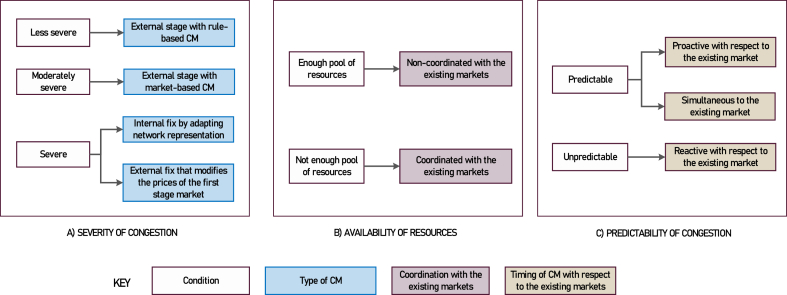
Observed relationship between a) severity of congestion, b) availability of resources c) predictability of congestion, and the type of CM method that is most suitable. Note that the relationship between settlement granularity and market clearing granularity is not considered here, as it depends on socio-political factors rather than quantifiable indicators.

When the severity of congestion is moderately high (but not high enough to demand zonal reconfiguration), a market-based mechanism is recommended to provide transparent locational signals for the investors. Conversely, if the severity of congestion is low (e.g., congestion due to an outage of a component or unavailability of certain generators due to maintenance), a rule-based mechanism might be sufficient, as locational signals are not very significant in those cases. In both market-based and rule-based procurement, the need to consider the non-dispatched bids within the existing market for CM is mainly determined by the availability of a large enough pool of bids for independent competitive procurement.^15^ A non-coordinated CM market is a good option if a competitive independent pool of bids is available for CM. Conversely, a coordinated CM market may be efficient if competitive procurement from separate CM bids is not an option. The last design option, the timing of CM relative to that of the existing market, should be considered in light of the predictability of the congestion. If the congestion in a system is predictable and geographically stable, then TSO can proactively or simultaneously manage the congestion. When congestion cannot be accurately predicted, TSO requires having available the market results to conduct the congestion assessment and address the identified congestion. The pattern is summarized in Fig. 10.

The timing of the European intraday and balancing markets are being shifted so that they run closer to real-time. This is mainly due to the integration of growing amounts of renewable generation and demand response resources [72]. Hence, proactive congestion management methods, such as bid filtering, are gaining more and more popularity among TSOs. However, these CM processes are plagued with a lack of transparency. Even if congestion is predictable, applying a proactive CM approach would involve incurring some efficiency losses. Besides, they can be deemed unfair by those agents who are continuously being filtered out. The best option for partially unpredictable congestion is to integrate the congestion management into the market, i.e., allocate capacity based on a security constrained program. Given the implementation challenges faced by such approaches in Europe, possible alternatives to proactive bid filtering include, among others, implementing a capacity market for congestion management so that the TSOs have access to fast-responding CM resources close to real-time.

## 5 Conclusions and policy implications

At the national level, and when considered in isolation from other markets, the different CM methods used in different European countries may deliver the results that they are intended to deliver. However, when embedded inside decentralized sequential markets, the CM markets can have unintended interactions with the already coupled wholesale markets. In addition to these seam issues, the lack of regulatory harmonization and consistent signals across Europe can impact investment opportunities. One can argue that intrazonal CM is a local system service that should be maintained within the national scope. While this argument is somewhat valid, the lack of transparency regarding the functioning of intrazonal CM cannot be justified. EU regulations advocate for fair, non-discriminatory, transparent market-based intrazonal CM wherever possible. Mapping intrazonal congestion designs, similar to ENTSO-E's publication of balancing market designs, can be a good starting point for achieving this goal. The availability of data on the intrazonal CM methods can facilitate knowledge exchange between TSOs, and foster convergence in market design.

Zonal markets are designed for power systems with abundant intrazonal transmission capacity and limited intrazonal congestion. However, at the pace at which the grid is developing, network investments and reinforcements may fall short, creating inefficient market dispatch. When network congestion leads to a highly inefficient market functioning, adaptation of the network model used in the market clearing is inevitable, whether through zonal reconfiguration or a shift to a nodal design. The evolution of markets in the United States is an example of this. Recent publications by European organizations indicate that the EU is no longer taking a defensive stance in the ongoing nodal-zonal debates but rather becoming more open to implementing more granular markets, including an LMP-based system as a second-stage market within the coupled European markets [41,47]. Leveraging existing zonal reconfiguration processes for testing the implementation of more granular CM schemes is a possible way to accomplish this.

While more granular market designs are needed to accurately represent congestion and provide precise locational signals, a competitive, market-based, independent procurement of intrazonal CM resources is the best option if the implementation costs of a more granular design outweigh its benefits. Redispatch markets have a bad reputation in academic publications due to the gaming possibilities and high costs they render. Besides, the aggregation of congestion from all preceding markets and its management in the balancing timeframe within this market can indeed create inaccurate high price signals and increase the costs of CM, which would further generate negative implications for market efficiency and participation incentives. However, employing the prices computed in the redispatch market (which are actually the prices corresponding to the marginal generation dispatched in the redispatch market) to modify the prices of the existing first-stage markets would reduce the gaming incentives in the existing market and enhance the locational value of the price signals. A close assessment of the market sequence and pricing systems should be considered to reduce the CM costs and real-time redispatch needs.

Rule-based CM methods can be cost-effective but would not provide transparent locational signals driving generation/demand and network investments. This could lead to a situation where congestion that should be avoided through further system development persists. Also, if kept unchecked, rule-based mechanisms can lead to significant renewable curtailment since they normally do not incentivize the efficient location of new demand, storage, or generation. Hence, acknowledging the reasons for adopting a rule-based mechanism, higher transparency regarding the rules and outcomes, and the implementation of efficient prices resulting from the final dispatch whenever possible should be mandated wherever they are used.

## Data availability

Data sharing not applicable to this article as no datasets were generated or analyzed during the current study. Our work proceeds within a theoretical approach.

## CRediT authorship contribution statement

**Shilpa Bindu:** Writing – original draft, Visualization, Methodology, Investigation, Formal analysis, Conceptualization. **Luis Olmos:** Writing – review & editing, Writing – original draft, Validation, Supervision, Methodology, Conceptualization. **José Pablo Chaves Ávila:** Writing – review & editing, Validation, Supervision, Project administration, Methodology, Funding acquisition, Conceptualization.

## Declaration of competing interest

The authors declare that they have no known competing financial interests or personal relationships that could have appeared to influence the work reported in this paper.

## Footnotes

1 The congestion of lines that are significantly used by the cross-border transactions can be grouped with the cross-zonal congestions, even if the congested transmission line is within a zone.

2 Long-term CM is normally managed through transmission rights.

3 Unlike the efficiency features and the assessment criteria related to them, which are universally applicable, the criteria defined under the ease of implementation of a method should depend on the geographical, institutional, political and regulatory context of the market. In our case, all the criteria we have defined are relevant to European markets. Thus, the criterion 'compatibility with cross-border integration' is explicitly assessed due to the significance of cross-border integration in Europe. If the assessment is to be replicated elsewhere, it is imperative to carefully evaluate and deduce the relevant qualities for that specific market.

4 Note that Financial Transmission Rights (FTRs) can be used to provide some hedge from locational price differences to small-to-medium-size consumers not being able to properly manage the corresponding price risk. This can be achieved by providing these consumers for free with FTRs defined between their respective nodes/zones and the national balancing one. The value of these FTRs would correspond to a foregone revenue for the system from congestion fees that the authorities may be willing to waive.

5 Italy has seven bidding zones, but because of the use of unique national price (PUN), all the consumers pay the same price for electricity. Additionally, the balancing market has a central-dispatching model that clears the market, considering the network with a high level of detail, in a security constrained economic dispatch program. However, the program determines price at zonal level, with a single national price for consumers. Thus, Italy has a unique overlap of two methods discussed.

6 Here, we consider a market-based mechanism as any standalone market that trades congestion management products and provides price signals.

7 The CM stage requires more granular locational information than the existing markets. If bids from the existing market are used for CM, this information can be made available within the bid, as either mandatory or voluntary information to provide, depending on the approach, or separately from the bid, such as within the registration data.

8 We consider the market timing in an auction market as the time between the gate closure time (GCT) and the publication of results, and in a continuous trading market as the trading period.

9 In this paper, the word ‘reactive’ refers to the placement of congestion management with respect to the market clearing of the existing markets. This has to be separated from the emergency congestion management used by the TSO when a congestion occurs in real-time.

10 The number of nodes represented in a model does not necessarily equal the number of connection points. Such simplifications can also be seen in the number of nodes considered for pricing. For instance, PJM has 86,397 electrical nodes, of which only 10,000 are priced in the market [60].

11 However, if discussed and justified, there should not be any relevant impact of zone reconfiguration on the implementation of coordinated or integrated markets as long as this reconfiguration of zones is affecting all the interrelated markets and the number of zones defined is still limited. Only if the implementation of zones only concerns some markets, or the new number of zones defined is very high, the coordination among markets would become challenging and a full integration of national markets would become necessary.

12 This is nothing but a nodal pricing market. If a redispatch is considered with all the participants in the primary market, the whole setup will be a zonal market followed by a nodal market. In that case, the best way to generate locational signal is just to implement the nodal market alone. Additionally, such a scheme would be very similar to the CM methods discussed in Branch.

13 Even in central-dispatch balancing markets, where the TSO has better coordination of the system, the objective of day-ahead congestion management is to manage congestion while minimizing the deviations from the energy market positions.

14 The metric for the severity of congestion depends on the system. In zonal markets of California, severity was measured by Wepex's five percent rule. If the cost of congestion management in a year is more than 5 percent of the access charge times the capacity of the rated path, the congestion is severe [71]. In Europe, the CACM guidelines define severe congestion or structural congestion as “predictable, unambiguously defined, geographically stable and frequently recurring under normal power system conditions” [3]. This definition is quite vague and is open for interpretation.

15 Here, we are considering only cases with no capacity reservation. If a competitive pool is not available, the TSO can proactively procure capacity and use it for real-time activation. In such case, there is market coordination, but between capacity and energy markets for congestion management.
